# Fluctuations in annual climatic extremes are associated with reproductive variation in resident mountain chickadees

**DOI:** 10.1098/rsos.171604

**Published:** 2018-05-09

**Authors:** Dovid Y. Kozlovsky, Carrie L. Branch, Angela M. Pitera, Vladimir V. Pravosudov

**Affiliations:** Department of Biology and Ecology, Evolution and Conservation Doctoral Program, University of Nevada, Reno, NV, USA

**Keywords:** climate change, reproduction, chickadee, elevation, snow, drought

## Abstract

Mounting evidence suggests that we are experiencing rapidly accelerating global climate change. Understanding how climate change may affect life is critical to identifying species and populations that are vulnerable. Most current research focuses on investigating how organisms may respond to gradual warming, but another effect of climate change is extreme annual variation in precipitation associated with alternations between drought and unusually heavy precipitation, like that exhibited in the western regions of North America. Understanding climate change effects on animal reproductive behaviour is especially important, because it directly impacts population persistence. Here, we present data on reproduction in nest-box breeding, resident mountain chickadees inhabiting high and low elevations in the Sierra Nevada across 5 years. These 5 years of data represent the full range of climatic variation from the largest drought in five centuries to one of the heaviest snow years on record. There were significant differences in most reproductive characteristics associated with variation in climate. Both climate extremes were negatively associated with reproductive success at high and low elevations, but low-elevation chickadees had worse reproductive success in the largest drought year while high-elevation chickadees had worse reproductive success in the heaviest snow year. Considering that the frequency of extreme climate swings between drought and snow is predicted to increase, such swings may have negative effects on chickadee populations across the entire elevation gradient, as climatic extremes should favour different adaptations. Alternatively, it is possible that climate fluctuations might favour preserving genetic variation allowing for higher resilience. It is too early to make specific predictions regarding how increased frequency of extreme climate fluctuation may impact chickadees; however, our data suggest that even the most common species may be susceptible.

## 1. Introduction

There is growing evidence that we are experiencing rapidly accelerating climate change across the globe associated with anthropogenic activity. Documented climatic changes include: warmer ambient air temperatures [1–3], highly variable precipitation patterns [4,5], increasing drought frequency [1,6–8], reduced snow cover [9–13] and increased occurrences of severe weather events [8,14–18]. Changes in these climate components have the potential to have a significant impact on living organisms and on biodiversity, and it is critical to understand how different species and populations may cope with climatic changes and whether these coping strategies are driven by rapid adaptations or plasticity [19–21]. As the effects of climate change on organismal phenotypes are hard to establish directly, long-term field studies are a crucial tool to establish how species may respond to annual, seasonal and environmental climatic variation (e.g. [21–26]).

In birds, much of the research has focused on how variation in precipitation, temperature and snow affect reproduction in cavity nesting species (e.g. [26–40]). Understanding how climate may affect reproduction is especially important as reproduction has a direct impact on population persistence and dynamics. Many previous studies have resulted in equivocal findings for different reproductive parameters such as lay date, clutch size, fledging success and nestling mass. The disparity in results may be a function of variation in climate having different effects on reproduction depending on specific features of the environment and species ecology. This is perhaps unsurprising given that, in most instances, climate is thought to affect reproduction indirectly, through its effect on food availability [27].

Since climatic norms and variation differ across environments, it is important to study the effects of variation in climate in a wide range of ecosystems to address spatial and temporal variation in climate [41] and how such variation may be associated with differences in reproduction. Most previous studies have focused on continuing trends in climate warming [42] and on singular or unidirectional extreme weather events (e.g. [22,43–45]), but climate change is also associated with drastic swings in extreme weather patterns associated with droughts, alternating between extreme droughts and extreme precipitation events. While scientists are interested in both ends of climatic extremes (e.g. [46]), there is currently a sparsity of data on swings in climatic extremes. Such drought-related variation in climate is exhibited in the semi-arid mountains of western North America. In the Sierra Nevada Mountains, for instance, precipitation varies greatly from year to year [47,48]. Drought conditions have a strong potential to directly affect both plants and invertebrates, which in turn may indirectly affect reproduction in many other species, such as birds, which rely on invertebrates to raise young. The Sierra Nevada, and other semi-arid mountain ranges, are unique in that most of the moisture for the year falls in the form of snow, with more snow (and moisture) occurring at higher elevations and the snowpack at higher elevations driving spring and summer moisture at lower elevations [48]. Increased snow fall and longer snow cover at higher elevations can directly affect survival and reproductive success in many species; therefore, it is likely that climate variation may affect populations differentially along an elevation gradient. Populations/species at low elevations can be expected to suffer more from drought owing to reduced direct precipitation and reduced indirect moisture running off from high elevations, but populations at higher elevations can be expected to suffer more from extreme winter climate, owing to severe snow fall and snowpack shortening the breeding window and reducing invertebrate abundance.

Here, we report 5 years of data on cavity nesting, resident mountain chickadees (*Poecile gambeli*) breeding in nest-boxes at high and low elevations in the Sierra Nevada. These 5 years of data are associated with the full range of drought-related variation in climate from the largest drought in five centuries [49] to the one of the heaviest snow years on record [50]. Such extreme variation, which typically would have occurred over the course of *ca* 100 years, has occurred within just 5 years, therefore providing a unique opportunity to investigate how one of the most common resident montane avian species in the Sierra Nevada may respond to an increased frequency of extreme climate swings.

## 2. Material and methods

### 2.1. Study subjects and field site

Mountain chickadees are resident passerine birds that store food during food surpluses in late summer and autumn to survive winter when food is scarce [51,52]. Mountain chickadees use a mixture of conifer seeds and invertebrates during the non-breeding season, but switch almost entirely to arthropods (mainly adult insects, insect larvae and spiders) during the breeding season. Nests are constructed within pre-existing cavities, but mountain chickadees also readily use nest-boxes for breeding [51].

All fieldwork was conducted at Sagehen Experimental Forest, Tahoe National Forest, approximately 14.5 km north of Truckee, CA, USA, at our long-term low (approx. 1900 m) and high (approx. 2400 m) elevation field sites during the 2013–2017 breeding seasons (May–August). At our field site, we maintain over 300 nest-boxes (more than 150 at each elevation) that we have been monitoring for the last five breeding seasons. In addition to nest-boxes, there are three snow telemetry (SNOTEL) (https://www.wcc.nrcs.usda.gov/snow/) weather stations (one at high elevation—directly in the middle of our sampling site, and two at low elevation—4.5 and 6 km from our site at the same elevation) that provide detailed weather data.

### 2.2. Climate data

Five years of our study have been associated with a full range of drought-related climatic variation, ranging from the largest drought in five centuries (2014–2015; [49]) to one of the heaviest snow years on record (2016–2017; [50]). Of the 3 years between the extremes, one was an average climate year (2015–2016), one was a slightly below average year (2012–2013) and one was slightly less dry than the record drought year (2013–2014). The climatic differences were most pronounced during the winter and spring months.

All climate data were obtained from the three SNOTEL weather stations near our field sites. SNOTEL stations are weather stations in western North America supported by the National Water and Climate Service (a branch of the United States Department of Agriculture's Natural Resources and Conservation Service). SNOTEL 541—Independence Lake (approx. 2500 m; https://wcc.sc.egov.usda.gov/nwcc/site?sitenum=541) was used for high-elevation climate data, and SNOTEL 540—Independence Camp (approx. 2100 m; https://wcc.sc.egov.usda.gov/nwcc/site?sitenum=540) and SNOTEL 539—Independence Creek (approx. 1950 m; https://wcc.sc.egov.usda.gov/nwcc/site?sitenum=539) were used for low-elevation climate data. From each SNOTEL station, we extracted daily mean temperatures (°C), precipitation (cm) and snow depth (cm). Data on precipitation and snow depth were originally in imperial units and were converted to metric units for analyses. We calculated monthly and yearly means associated with each year of the breeding data using daily mean data. Monthly and yearly means for each breeding season began on 1 September of the previous year and ended on 31 August of that breeding season.

Yearly mean snow depth was used to rank each year in terms of drought level because much of the precipitation in the Sierra Nevada Mountains is in the form of snow (this is especially true for high elevations; [48]), and snowmelt run-off from higher elevations in montane environments is often a direct driver of breeding season moisture at lower elevations (e.g. [53]).

### 2.3. Nesting data

Only nest-boxes were used for the current study and as such we have no breeding data on chickadees breeding in natural cavities. All nest-boxes were monitored for nest building beginning on the first couple days of May each year. Nests were monitored several times a week until eggs were detected. When multiple eggs were detected, we calculated the start of egg laying based on the assumption that chickadees produce one egg per day [51]. Once the final clutch size was calculated, the beginning of incubation was determined as the day following the last egg being laid. As chickadee eggs hatch approximately 13 days after incubation has begun (e.g. [54]), we began checking for hatching at that time. We determined pre-fledging brood size 15 days after we detected any hatchlings, and all nestlings were banded with United States Geological Survey aluminium bands and weighed on that day. We also calculated the within-nest coefficient of variation (CV) for pre-fledging mass for each nest. We only used first nesting attempts in the current study (regular second clutches following successful completion of the first reproduction were observed only at low elevation during the average climate year—2016). Overall, we had 425 nests that began laying—165 nests at high elevation (21, 34, 49, 32 and 29 nests consecutively during each of the 5 years) and 260 nests at low elevation (34, 63, 57, 42 and 64 nests consecutively during each of the 5 years). Some of these nests dropped out at various stages of the breeding season owing to egg or nestling mortality.

### 2.4. Statistical analysis

All data were analysed in R statistical software [55] using linear models and linear mixed models using year as a random variable. ANOVA tables were generated using the car package [56] and post hoc analyses used lsmeans [57] and multcomp packages [58]. Weather was analysed with one weather parameter (snow depth, precipitation or mean temperature) as the response variable and year, month and site as predictor variables. For breeding, we analysed all breeding parameters (Julian first egg date, clutch size, pre-fledging brood size, pre-fledging nestling mass or the CV for pre-fledging mass) with mean monthly snow depth from January to June (as this represents the period where weather is most likely to impact chickadee reproduction in our system) and elevation as fixed effects using year as a random effect. We additionally analysed breeding data using each breeding parameter as the response variable and year and elevation (both categorical) used as predictor variables. This second set of analyses is important to address potential carry over and compounding effects of climatic conditions across time. In addition, each year was associated with a unique climate pattern, and comparing reproduction among years ranging between the two opposite extreme climate patterns is highly informative for understanding how annual variation in all combined climate conditions might have influenced reproduction in chickadees at different elevations. Analyses were run with and without biologically relevant covariates (e.g. Julian first egg date for an analysis of clutch size and clutch size for an analysis of brood size; see results below). In the analyses of yearly variation, each year (used as a categorical variable) was treated as an independent event as we do not have banding data on all parents during all years, which prevents analysing differences within individuals.

## 3. Results

### 3.1. Weather

Month was a significant predictor of precipitation, and the interactions between year and month, month and elevation, and the three-way interaction between year, month and elevation were significant (table 1). High elevation had significantly more precipitation than low elevation and 2017 had significantly more precipitation than all other years (Tukey's HSD: all *p* < 0.05).

**Table 1.** *F-*statistics for models with weather parameters as a response variable and year, month and site as predictor variables. Italic statistics are statistically significant at *α* = 0.05.

model	parameter	*F-*statistics
precipitation	year	*F_5_*_,*3631*_* *=* 195.00*, *p *<* 0.001*
	month	*F_11_*_,*3631*_* *=* 330.87*, *p *<* 0.001*
	site	*F_1_*_,*3631*_* *=* 210.88*, *p *<* 0.001*
	year × month	*F_42_*_,*3631*_* *=* 238.28*, *p *<* 0.001*
	year × site	*F_5_*_,*3631*_* *=* 38.98*, *p *<* 0.001*
	month × site	*F_11_*_,*3631*_* *=* 28.39*, *p *<* 0.001*
	year × month × site	*F_42_*_,*3631*_* *=* 16.95*, *p *<* 0.001*
snow depth	year	*F*_5,3532_ = 0.019, *p* > 0.9
	month	*F_11_*_,*3532*_ = *812.53*, *p *<* 0.001*
	site	*F*_1,3532_ = 0.00, *p* < 0.001
	year × month	*F_44_*_,*3532*_ = *812.53*, *p *<* 0.001*
	year × site	*F*_5,3532_ = 0.0093, *p* > 0.9
	month × site	*F_11_*_,*3532*_* *=* 295.21*, *p *<* 0.001*
	year × month × site	*F_42_*_,*3532*_* *=* 132.20*, *p *<* 0.001*
mean temperature	year	*F_5_*_,*3644*_* *=* 34.20*, *p *<* 0.001*
	month	*F_11_*_,*3644*_* *=* 397.52*, *p *<* 0.001*
	site	*F_1_*_,*3644*_* *=* 3.96*, *p *=* 0.02*
	year × month	*F_42_*_,*3644*_* *=* 108.08*, *p *<* 0.001*
	year × site	*F*_5,3644_ = 0.29, *p* = 0.59
	month × site	*F*_11,3644_ = 0.016, *p* = 0.90
	year × month × site	*F*_42,3644_ = 0.77, *p* = 0.38

**Figure 1.**
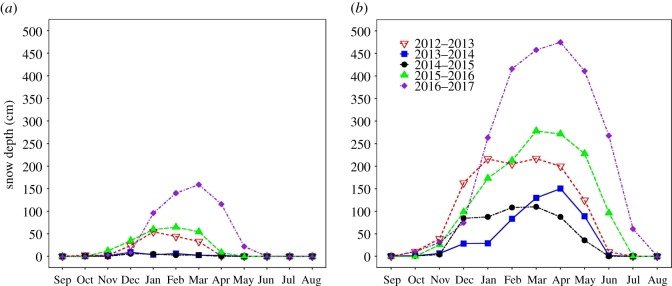
Monthly snow depth for (*a*) low and (*b*) high elevations for 5 years (2012–2017). Each year starts on 1 September and ends on 31 August.

Snow depth varied significantly with year, month and elevation, and all interactions were significant (table 1). Similar to precipitation, high elevation had significantly more snow across all years and 2017 was associated with significantly more snow than all other years (Tukey's HSD: all *p* < 0.001; figure 1). Both 2013–2014 and 2014–2015 had significantly less snow than other years (Tukey's HSD: all *p* < 0.001), but the amount of snow within those 2 years was not significantly different (Tukey's HSD: *p* = 0.99).

**Figure 2.**
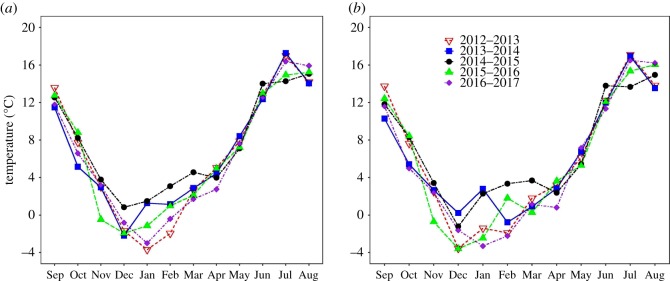
Monthly mean temperature for (*a*) low and (*b*) high elevations for 5 years (2012–2017). Each year starts on 1 September and ends on 31 August.

Year, month and elevation were also significant predictors of mean daily temperature (table 1 and figure 2). The interaction between year and month was also significant; however, the interactions between year and elevation, month and elevation and between year, month and elevation were not statistically significant (table 1 and figure 2). Additionally, we found a significant negative correlation between snow depth and mean temperature regardless of elevation (*r*^2^ = 0.67, *p* = 0.003)—years associated with more snow were also associated with significantly lower ambient temperature.

### 3.2. Annual breeding

#### 3.2.1. First egg date

**Figure 3.**
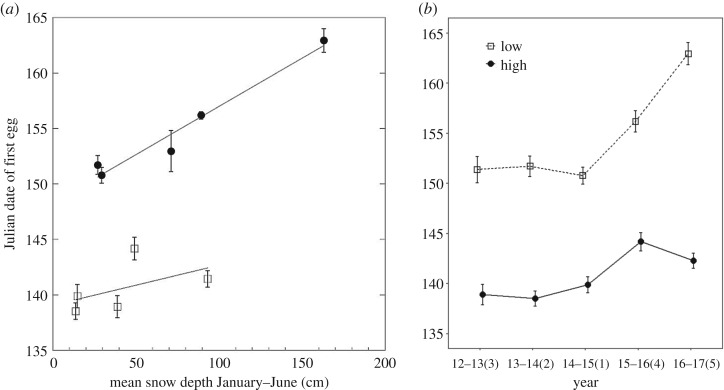
(*a*) The adjusted Julian date (starts over each year) of the first egg for low (squares) and high (circles) elevation chickadees and mean monthly snow depth from January to June (means ± s.e.). A smoothing function was added separately for each elevation. (*b*) The adjusted Julian date (starts over each year) that the first egg was laid for low (squares, dashed line) and high (circles, solid line) elevation chickadees across 5 years of breeding. The *x*-axis labels are years with ranks by mean yearly snow depth going from 1 (least snow) to 5 (most snow) in parentheses (means ± s.e.).

When year was included as a random effect, snow depth was not a significant predictor of the date the first egg was laid (*Wald χ*^2^ = 3.11, *p* = 0.08); however, elevation was a significant predictor (*Wald χ*^2^ = 82.75, *p* < 0.001) and there also was a significant interaction between snow depth and elevation (*Wald χ*^2^ = 5.42, *p* = 0.02). Snow depth appears to be associated with the first egg laying date specifically at high elevation where more snow was associated with later egg laying, while at low elevation this pattern was much less pronounced (figure 3*a*).

Chickadees at high elevation started laying significantly later during all years than birds at low elevations, and birds from both elevations started laying later in years with more snow, albeit the differences in timing between drought and snow years were larger specifically at high elevation (figure 3*b* and table 2).

**Table 2.** General linear model results testing for differences among years for all breeding parameters without (above) and with (below) covariates included (when applicable). Response variables are in columns and predictor variables are across rows. Italic statistics indicate significant results at *α* = 0.05.

model	parameter	lay date	clutch	pre-fledging brood	pre-fledging mass	mass CV
without covariate						
	*year*	*F_4_*_,*415*_* *=* 7.75*, *p *<* 0.001*	*F_4_*_,*367*_* *=* 17.70*, *p *<* 0.001*	*F_4_*_,*295*_* *=* 6.99*, *p *<* 0.001*	*F_4_*_,*294*_* *=* 12.69*, *p *<* 0.001*	*F_4_*_,*294*_* *=* 4.53*, *p *=* 0.008*
	*elevation*	*F_1_*_,*415*_* *=* 56.12*, *p *<* 0.001*	*F_1_*_,*367*_* *=* 4.35*, *p *=* 0.04*	*F*_1,295_ = 0.89, *p* = 0.34	*F*_1,294_ = 1.35, *p* = 0.25	*F*_1,294_ = 0.064, *p* = 0.80
	*interaction*	*F_4_*_,*415*_* *=* 8.67*, *p *<* 0.001*	*F_4_*_,*367*_* *=* 8.64*, *p *<* 0.001*	*F_4_*_,*295*_* *=* 5.39*, *p *<* 0.001*	*F_4_*_,*294*_* *=* 4.76*, *p *<* 0.001*	*F*_4,294_ = 1.52, *p* = 0.20

			covariate: lay date	covariate: clutch	covariate: lay date	
with covariate						
	*year*		*F_4_*_,*366*_* *=* 25.33*, *p *<* 0.001*	*F*_4,3294_ = 1.17, *p* = 0.33	*F_4_*_,*291*_* *=* 11.70*, *p *<* 0.001*	
	*elevation*		*F_1_*_,*366*_* *=* 17.18*, *p *=* 0.001*	*F*_1,294_ = 0.34, *p* = 0.56	*F*_1,291_ = 0.09, *p* = 0.76	
	*interaction*		*F_4_*_,*366*_* *=* 5.87*, *p *<* 0.001*	*F_4_*_,*294*_* *=* 2.93*, *p *=* 0.02*	*F_4_*_,*291*_* *=* 5.30*, *p *<* 0.001*	
	*covariate*		*F_1_*_,*366*_* *=* 33.89*, *p *<* 0.001*	*F_1_*_,*3294*_* *=* 158.49*, *p *<* 0.001*	*F_1_*_,*291*_* *=* 15.77*, *p *<* 0.001*	

#### 3.2.2. Clutch size

**Figure 4.**
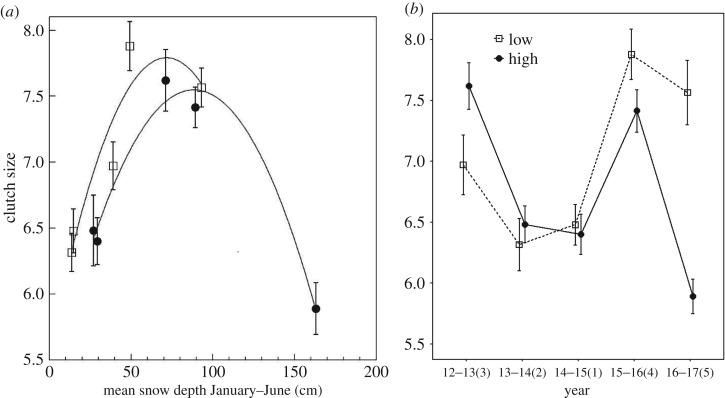
(*a*) Clutch size for low (squares) and high (circles) elevation chickadees and mean monthly snow depth from January to June (means ± s.e.). A smoothing function was added separately for each elevation. (*b*) Clutch size for low (squares, dashed line) and high (circles, solid line) elevation chickadees across 5 years of breeding. The *x*-axis labels are years with ranks by mean yearly snow depth going from 1 (least snow) to 5 (most snow) in parentheses (means ± s.e.).

When year was included as a random effect, clutch size was significantly associated with snow depth and elevation, and the interaction between snow depth and elevation was statistically significant (*Wald χ*^2^ = 3.75, *p* = 0.05; *Wald χ*^2^ = 6.31, *p* = 0.01; *Wald χ*^2^ = 16.14, *p* < 0.001; respectively; figure 4*a*). Lay date was significantly and negatively associated with clutch size when it was included as a covariate, but results remained similar with snow depth, elevation and the interaction between snow levels and elevation remaining significant predictors (all *p* < 0.03) with a significant interaction between elevation and first egg date (*Wald χ*^2^ = 8.26, *p* = 0.004). There was an inverted U-shaped relationship between clutch size and snow depth at high elevation, with smaller clutches associated with more snow and larger clutches associated with the average snow depth. At low elevation, the smallest clutches were observed during the lowest snow depth (e.g. drought), and the largest clutches during both the average and the extremely high snow depth (figure 4*a*).

While high-elevation chickadees had the smallest clutches during both the driest (2014–2015) and snowiest (2016–2017) years, low-elevation chickadees had the smallest clutches only during the driest year (2014–2015; figure 4*b* and table 2). During the snowiest year (2016–2017), low-elevation chickadees had clutch sizes similar to those during the average snow year (2015–2016) (Tukey's HSD: *p* = 0.93) and larger than clutches of chickadees at high elevation (Tukey's HSD: *p* < 0.001).

Lay date was significantly and negatively associated with clutch size when included as a covariate (table 2), but the yearly differences followed a similar pattern, except high-elevation chickadees had significantly larger clutches during the slightly below average snow year (2012–2013; Tukey's HSD: *p* = 0.0015), and the difference in clutch size between high- and low-elevation chickadees during the snowiest year (2016–17) became non-significant (Tukey's HSD: *p* = 0.81).

#### 3.2.3. Brood size

**Figure 5.**
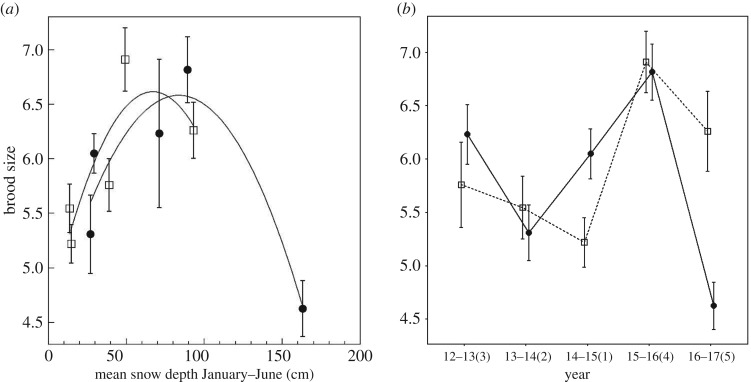
(*a*) Unadjusted brood size for low (squares) and high (circles) elevation chickadees and mean monthly snow depth from January to June (means ± s.e.). A smoothing function was added separately for each elevation. (*b*) Unadjusted brood size for low (squares, dashed line) and high (circles, solid line) elevation chickadees across 5 years of breeding. The *x*-axis labels are years with ranks by mean yearly snow depth going from 1 (least snow) to 5 (most snow) in parentheses (means ± s.e.).

When year was included as a random effect, snow depth was not a significant predictor of overall brood size (*Wald χ*^2^ = 1.77, *p* = 0.18), but elevation differences were significant (*Wald χ*^2^ = 8.30, *p* = 0.004) and the interaction between snow depth and elevation was also statistically significant (*Wald χ*^2^ = 9.95, *p* = 0.002). Like clutch size, smaller brood size at low elevation was observed in years with extremely low snow depth (e.g. drought) while the largest broods were observed in years with more snow (figure 5*a*). At high elevation, brood size also followed an inverse U-shaped relationship with snow depth; smallest broods were observed during either extreme of snow depth (very little snow or extremely high amount of snow) and the largest broods during the year with an average snow depth (figure 5*a*).

When clutch size was included as a covariate, snow depth was not significantly associated with relative brood size (*Wald χ*^2^ = 0.0093, *p* = 0.92). Elevation was marginally, but not significantly, related to relative brood size (*Wald χ*^2^ = 3.47, *p* = 0.06), and the interaction between snow depth and elevation was not statistically significant (*Wald χ*^2^ = 0.75, *p* = 0.39). Clutch size, however, was a significant covariate (*Wald χ*^2^ = 175.11, *p* < 0.001). Therefore, although overall brood size was significantly associated with snow depth, chickadees appear to have produced the same brood size relative to the number of eggs laid across the years with different climate because clutch size and brood size varied similarly across years with different climatic conditions.

When comparing brood size across different years, high-elevation chickadees had significantly larger broods during the average climate year (2015–2016) than during the first drought year (2013–2014; Tukey's HSD: *p* = 0.036) and the extreme snow year (2016–2017; Tukey's HSD: *p* < 0.001; figure 5*b* and table 2). Low-elevation chickadees had significantly larger broods during the snowiest year (2016–2017) and average snow year (2015–2016) than during the worse drought year (Tukey's HSD: all *p* < 0.04). Compared with low-elevation chickadees, high-elevation chickadees tended to have larger broods relative to the clutch size during the worst drought year (2014–2015), but similar brood sizes during all other years (Tukey's HSD: all *p* > 0.1).

#### 3.2.4. Pre-fledging mass

**Figure 6.**
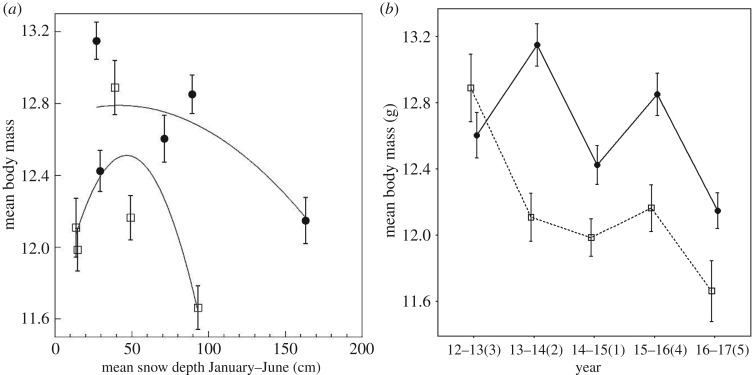
(*a*) Pre-fledging mass for low (squares) and high (circles) elevation chickadees and mean monthly snow depth from January to June (means ± s.e.). A smoothing function was added separately for each elevation. (*b*) Pre-fledging mass (taken at day 16 after hatching) for low (squares, dashed line) and high (circles, solid line) elevation chickadees across 5 years of breeding. The *x*-axis labels are years with ranks by mean yearly snow depth going from 1 (least snow) to 5 (most snow) in parentheses (means ± s.e.).

When year was included as a random effect, there were significant differences in pre-fledging mass between elevations (*Wald χ*^2^ = 17.15, *p* < 0.001), but snow depth and snow depth by elevation interaction were not statistically significant (snow depth: *Wald χ*^2^ = 0.86, *p* = 0.35; snow depth × elevation: *Wald χ*^2^ = 0.066, *p* = 0.80). First egg date was a significant covariate for mean pre-fledging mass (*Wald χ*^2^ = 13.09, *p* < 0.001), but results remained similar when the covariate was included (elevation: *p* < 0.001; snow depth: *p* = 0.44; interaction: *p* = 0.51). Regardless of snow depth, high-elevation chickadees fledged young with higher mass than low-elevation chickadees (figure 6*a*).

At both high and low elevations, lowest pre-fledging mass was recorded in the snowiest year (2016–2017; figure 6*b* and table 2); however, the differences were only significant between the snowiest year and the slightly below average snow year (2012–2013) for low elevation (Tukey's HSD: *p* < 0.0001, all remaining *p* > 0.9) and between the snowiest year and the second to worst drought year (2013–2014) for high elevation (Tukey's HSD: *p* < 0.01, all remaining *p* > 0.8; figure 6*b*).

Pre-fledging mass was significantly higher at high elevation than at low elevation during the second to worst drought year (2013–2014) and during the average snow year (2015–2016; Tukey's HSD: *p* < 0.05), but was not significantly different between elevations during the other years (Tukey's HSD: all *p*>0.1).

With lay date as a covariate, the yearly pattern in pre-fledging mass remained similar (table 2). However, the highest pre-fledging mass recorded for low-elevation chickadees during the slightly below average year (2012–2013) was significantly different from all other years with lay date as a covariate (Tukey's HSD: all *p* < 0.01) and high-elevation chickadees had significantly higher pre-fledging mass during all years, except the slightly below average snow year and the driest year (Tukey's HSD: all *p* < 0.01).

#### 3.2.5. Within-brood variation in pre-fledging mass (coefficient of variation)

**Figure 7.**
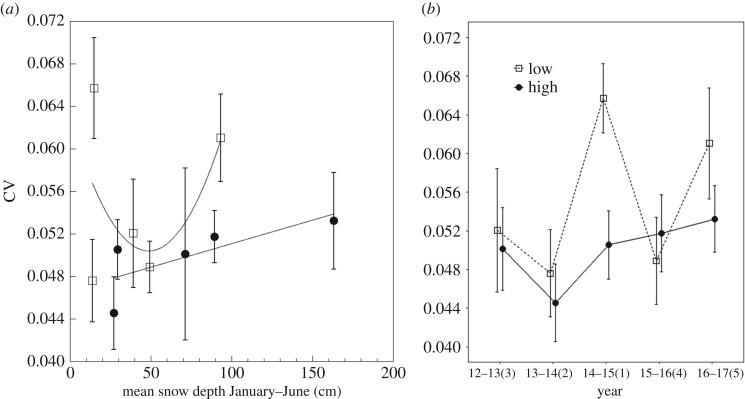
(*a*) CV for pre-fledging mass for low (squares) and high (circles) elevation chickadees and mean monthly snow depth from January to June (means ± s.e.). A smoothing function was added separately for each elevation. (*b*) CV for pre-fledging mass for low (squares, dashed line) and high (circles, solid line) elevation chickadees across 5 years of breeding. The *x*-axis labels are years with ranks by mean yearly snow depth going from 1 (least snow) to 5 (most snow) in parentheses (means ± s.e.).

When pre-fledging mass CV was analysed in a mixed effects model with year as a random effect, there were no significant effects of snow depth and elevation (*Wald χ*^2^ = 0.21, *p* = 0.64; *Wald χ*^2^ = 3.24, *p* = 0.07; respectively) and the snow depth by elevation interaction was also not statistically significant (*Wald χ*^2^ = 0.035, *p* = 0.85; figure 7*a*).

When analysing mass CV across different years, low-elevation chickadees had the highest within-brood variation during the most extreme years; during the record drought year, they had the highest CV compared with the other three between-extreme years, but this result was only significantly different when comparing the record drought with the second to the worst drought and with the average year (Tukey's HSD: *p* < 0.05; table 2). During the record drought year, low-elevation chickadees had a non-significant trend to have larger within-brood CV compared with high-elevation birds (Tukey's HSD: *p* = 0.079; figure 5). In contrast to low elevation, there were no significant differences in within-brood variation in mass among the 5 years at high elevation (Tukey's HSD: all *p* > 0.9).

## 4. Discussion

Our study shows substantial variation in reproduction associated with temporal and spatial climate variation in mountain chickadees inhabiting high and low elevations in Sierra Nevada. We found that almost all breeding parameters varied significantly among years and between high and low elevations, and that climate conditions on the drought–extreme snow scale were associated with different effects on chickadee reproduction between elevations. Low-elevation chickadees had the worst reproduction success (specifically clutch size and brood size) during the worst drought year, while high-elevation chickadees had the worst reproductive success during the largest snow year. At both elevations, the highest reproductive success was observed during the average snow year.

Clutch size and brood size were the main breeding parameters that varied significantly with yearly differences in drought-related climate at both elevations, yet the climate extremes seemed to have the opposite effects at different elevations. High-elevation chickadees exhibited an inverted U-shaped relationship with the largest clutches and broods in years with average and near average climate and smallest clutches and broods during the extreme snow year. Low-elevation chickadees had the smallest clutches and broods during the two driest years, but the largest clutches and broods during the average climate year and during the record snow year. These results suggest that drought has the most negative effect on chickadee reproduction at low elevation while extreme snow has the most negative effect at high elevation.

The overall reproductive output (the sheer number of fledglings produced) appeared to be highest for both elevations during the average snow year (2015–2016), lowest for high elevation during the record snow year (2016–2017) and lowest for low elevation during the record drought year.

In all but a single year, high-elevation chickadees reared more offspring relative to clutch size than low-elevation chickadees, which suggests that at high elevation snow levels may serve as a better predictor of conditions during young rearing than at low elevation. At low elevation, chickadees may use different cues to initiate breeding, potentially providing a mismatch between the start of reproduction and the emergence of abundant invertebrate food sources (e.g. [59]).

Climate variation is typically thought to affect reproduction through its effects on prey abundance [27]. While we do not have data on climate and invertebrate abundance for our field site, our results appear consistent with this hypothesis. During drought years, low-elevation chickadees would probably have less invertebrate-based food resources because of low direct moisture (in the form of rain and snow) and lower and shorter periods of indirect soil moisture coming from high elevations [60] directly affecting invertebrate abundance. Lower invertebrate density and availability could drive the results we see here for low-elevation chickadees in terms of smaller clutches (especially later in the season), smaller pre-fledging broods, lighter fledglings and larger within-brood variation in pre-fledging mass during drought years.

A mismatch between timing of breeding and food availability specifically at low elevation can also potentially explain smaller clutches, but similar to low-elevation brood sizes at high elevation during the severe snow year compared with other years. Smaller clutches may be related to starting breeding before the invertebrate prey populations have fully recovered following winter, but high-elevation chickadees might not be able to afford delaying breeding owing to their extremely narrow breeding window. Later in the season during nestling feeding, prey abundance may have been sufficient to allow the relatively few nestlings that were produced to be fed sufficiently. It is likely that high-elevation chickadees use snow levels as the main cue to start breeding, and it is also likely that snow conditions during that time serve as a good predictor of invertebrate abundance later during the summer. At the same time, at low elevation, it appears that extreme years provide a larger mismatch in conditions during egg laying and rearing young. Which additional cues birds use to determine the start of laying, besides day length and snow levels, remains an avenue for future research [39]. It is also possible that invertebrate abundance during extremely wet years is generally low at both elevations, but with different peak timings as increased precipitation and moisture above a certain threshold have been linked to reduced invertebrate abundance (e.g. [61]).

Another important result indicative of negative effects of drought specifically on low-elevation chickadees is within-brood variation in pre-fledging mass. Pre-fledging mass is well known to be critical in determining post-fledging survival and recruitment success (e.g. [62]), and, as such, variation in pre-fledging mass has important implications for reproductive success and fitness. At low elevation, within-brood variation in mass tended to be higher during the record drought year, which suggests that parents were not able to rear all young equally. An interesting question is whether in such cases one or both parents preferentially invest in some young based on some phenotypic characteristic or sex (e.g. mouth colour [63]). Indeed, simulated models predict preferential investment in certain offspring in environments that differ in quality (e.g. [64]). In addition to higher within-brood variation in pre-fledging mass at low elevation, the mean pre-fledging mass was also lower during the driest and snowiest years. At high elevation, pre-fledging mass, but not within-brood variation, was also lower during the extreme years, which again suggests that high-elevation chickadees produce a more optimal clutch size for the number of nestlings they are capable of fledging regardless of climatic conditions.

Surprisingly, high-elevation chickadees, in all but one year, had heavier fledglings than their low-elevation counterparts. While smaller mass prior to fledging is sometimes indicative of poorer condition and developmental stress (e.g. [65]), it does not seem likely that the differences in our case are due to nutritional deficits and stress between elevations, at least not during all years. Even during the average climate year when low-elevation chickadees did the best, they fledged chicks at lower mass than chickadees at high elevation. We think that these differences are more likely to be due to much later breeding at high elevation and the need to complete development and moult in a shorter time prior to winter conditions, which arrive earlier at high elevation. The exact mechanism allowing for higher body mass at high elevation, whether it is synchronicity between breeding and food, more abundance of food, greater parental care or some other factor, remains unknown.

Similarly, in a 5 year study of dark-eyed juncos along an elevation gradient, compared with lower elevations, nesting began later and nestlings were heavier at higher elevations while brood sizes were similar across elevations [39]. Compared with lower elevations, high-elevation juncos also survived longer, but had lower reproductive success each year, mainly because of multiple broods per season being attempted at low elevations, suggesting a slower life-history pace. While we do not currently have survival data for chickadees, our 5 years of breeding data showed no elevation-related differences in brood size and higher nestling mass at high elevation, but, unlike the study on juncos, low-elevation chickadees did not produce a second brood every year—we only detected second broods (after successful completion of the first reproductive event) at low elevation in one average climate year (2015–2016) and a couple of second broods at low elevation during the record snow year (2016–2017). Our data do, however, suggest that, during the ideal climate conditions, low-elevation chickadees are likely to be able to produce more offspring than high-elevation chickadees by rearing two broods.

One important caveat to our study is that all reproductive data were collected on nest-box breeding chickadees. It is possible that temperature, moisture or solar radiation differ between nest-boxes and natural cavities and that such differences could impact reproduction and that chickadees breeding in natural cavities may use different sites depending on the climate in a particular year. We, however, think that this is unlikely for a couple of reasons. Temperatures and moisture are often similar between nest-boxes and natural cavities (e.g. [66]), and nest-box (and natural cavity) orientation and the amount of foliage cover may impact both temperature and solar radiation (e.g. [66,67]). In the current study, both field sites had a tremendous abundance of unused natural cavities (V. V. Pravosudov, D. Y. Kozlovsky 2013–2017, personal observation), and if chickadees adjusted their nesting locations according to yearly climate, we would expect that, in some years, we would have many more used/unused nest-boxes. This, however, is not the case. The percentage of occupied nest-boxes is similar across all years and chickadees at our field sites seem to prefer breeding in nest-boxes rather than in natural cavities. Second, while some species show differences in reproduction between natural cavities and nest-boxes, others do not (e.g. [68]). We acknowledge that we cannot definitively say that nest-boxes had no effect on our data. However, it has become the standard to look at the association between environmental variables and reproduction in nest-box breeding species (e.g. [36,69–74]) because of the logistical constraints associated with natural nests.

While alternations of extreme climatic conditions are of interest to scientists studying the effects of climate change on various organisms (e.g. [46]), to our knowledge, previous empirical studies have only demonstrated that extreme weather events are associated with differences in animal phenotype (e.g. [22,43–45]). To our knowledge, no study has addressed how climatic extremes along a moisture–drought gradient may impact reproduction. These frequent alternations of extreme climate associated with climate change may have profound effects on the way selection may act on populations. Different climate extremes are likely to favour different phenotypic traits and rapid alternation of opposite extremes may result in maintaining high genetic variation due to frequent temporal shifts in climate, even when selection may drive a reduction in genetic variation at each climatic extreme [41]. For example, in the Sierra Nevada Mountains, drought years might favour certain phenotypes that are particularly well suited to those drought conditions; however, if the following year is an average or severe moisture year, directional selection may favour an entirely different suite of phenotypes. Considering that there is at least some gene flow between elevations in mountain chickadees [75] and indirect evidence for local adaptations (e.g. more food caching and better spatial memory associated with more caching at high elevation; [76,77]), rapid alternation of extreme climate may maintain more genetic variation, which in turn may make chickadees more resilient to climate change (e.g. [41]).

Overall, our study suggests that there are large negative effects of extreme climate variation on one of the most common resident species in the Sierra Nevada and such effects seemed to differ along an elevation gradient. Lower elevations seem to be most negatively affected by extreme droughts, while higher elevations appear to be most negatively affected by extreme snow levels. At this point, it is hard to predict how these climatic extremes may impact populations and species owing to the fact that these extremes occurred previously only once in a century, but may now become more and more frequent [8,14–18]. This may be especially true for montane species, because of the difficulty associated with making broad-scale predictions of precipitation patterns in these regions [78,79]. These results call attention to the need for studies of even the most common species, as sharp declines may occur as extreme climate variation becomes more frequent. Only with long-term monitoring of many species under both temporal and spatial variation in climatic extremes will we begin to understand the many effects of extreme climate fluctuation

## Supplementary Material

Weather Data

## Supplementary Material

Breeding Raw Data

## Data accessibility

All weather and breeding data are included in the electronic supplementary material.
