# Correction to: ‘Fluctuations in annual climatic extremes are associated with reproductive variation in resident mountain chickadees’

**DOI:** 10.1098/rsos.181248

**Published:** 2018-08-22

**Authors:** Dovid Y. Kozlovsky, Carrie L. Branch, Angela M. Pitera, Vladimir V. Pravosudov

*R. Soc. open sci.* **5**, 171604. (Published 9 May 2018). (doi:10.1098/rsos.171604)

There is an error in figure 3*b* in the published paper. The original graph had the incorrect labels associated with the data for high and low elevation lay dates. The corrected graph fixes this problem such that high elevation (black circles, solid line) and low elevation (open squares, dashed line) are now associated with the correct data. All high elevation lay dates were later in the season (larger Julian date values) than all low elevation lay dates. The corrected figure 3*b* is shown below.

**Figure 3.**
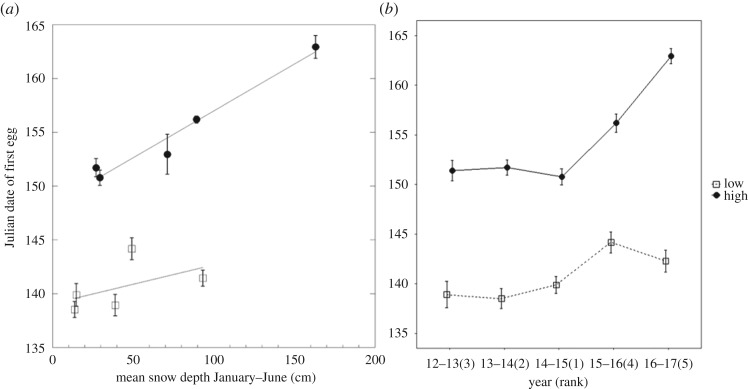
(*a*) The adjusted Julian date (starts over each year) of the first egg for low (squares) and high (circles) elevation chickadees and mean monthly snow depth from January to June (means ± s.e.). A smoothing function was added separately for each elevation. (*b*) The adjusted Julian date (starts over each year) that the first egg was laid for low (squares, dashed line) and high (circles, solid line) elevation chickadees across 5 years of breeding. The *x*-axis labels are years with ranks by mean yearly snow depth going from 1 (least snow) to 5 (most snow) in parentheses (means ± s.e.).

